# Role of TLR4-p38 MAPK-Hsp27 signal pathway in LPS-induced pulmonary epithelial hyperpermeability

**DOI:** 10.1186/s12890-018-0735-0

**Published:** 2018-11-27

**Authors:** Weiju Wang, Jie Weng, Lei Yu, Qiaobing Huang, Yong Jiang, Xiaohua Guo

**Affiliations:** Department of Pathophysiology, Guangdong Province Key Laboratory for Shock and Microcirculation Research, Guangdong Provincial Key Laboratory of Proteomics, State Key Laboratory of Organ Failure Research, Southern Medical University, Guangzhou, 510515 China

**Keywords:** ALI, Alveolar barrier dysfunction, P38 MAPK, LPS, TLR4, Hsp27, Cytoskeletal rearrangement

## Abstract

**Background:**

The breakdown of alveolar barrier dysfunction contributes to Lipopolysaccharide stimulated pulmonary edema and acute lung injury. Actin cytoskeleton has been implicated to be critical in regulation of epithelial barrier. Here, we performed in vivo and in vitro study to investigate role of TLR4-p38 MAPK-Hsp27 signal pathway in LPS-induced ALI.

**Methods:**

For in vivo studies, 6–8-week-old C57 mice were used, Bronchoalveolar lavage Fluid /Blood fluorescent ratio, wet-to-dry lung weight ratio, as well as protein concentrations and neutrophil cell counts in BALF were detected as either directly or indirectly indicators of pulmonary alveolar barrier dysfunction. And hematoxylin and eosin staining was performed to estimate pulmonary injury. The in vitro explorations of transepithelial permeability were achieved through transepithelial electrical resistance measurement and testing of FITC-Dextran transepithelial flux in A549. In addition, cytoskeletal rearrangement was tested through F-actin immunostaining. And SB203580 was used to inhibit p38 MAPK activation, while siRNA was administered to genetically knockdown specific protein.

**Results:**

We showed that LPS triggered activation of p38 MAPK, rearrangement of cytoskeleton which resulted in severe epithelial hyperpermeability and lung edema. A549 pretreated with TLR4 siRNA、p38 MAPK siRNA and its inhibitor SB203580 displayed a lower permeability and fewer stress fibers formation after LPS stimulation, accompanied with lower phosphorylation level of p38 MAPK and Hsp27, which verified the involvement of TLR4-p38 MAPK-Hsp27 in LPS-evoked alveolar epithelial injury. Inhibition of p38 MAPK activity with SB203580 in vivo attenuated pulmonary edema formation and hyperpermeability in response to LPS.

**Conclusions:**

Our study demonstrated that LPS increased alveolar epithelial permeability both in vitro and in vivo and that TLR4- p38 MAPK- Hsp27 signal pathway dependent actin remolding was involved in this process.

## Background

Acute lung injury (ALI) is a clinical syndrome that still remains high (30–40%) rates of mortality in spite of the great advances in mechanism research and therapy [1–3]. Among all the causes, Lipopolysaccharide (LPS), the outer layer of the most Gram-negative bacteria, has been wildly studied [4–6]. It has been frequently implicated that exposure to LPS leads to activation of various signal pathways, production of inflammatory mediators, and endothelial barrier dysfunction [7–9]. All these effects facilitate transcelluar permeability and lead to severe edema during ALI. In addition, accumulated evidences point to a role for alveolar epithelial barrier dysfunction during development of ALI [10–12]. However, the precise mechanism responsible for alveolar epithelial injury in LPS-induced ALI remains to be elusive, which is major theme of this study.

Zhijie et al. have performed an in-vivo study to determine the critical role of Toll-like receptor 4 (TLR4) in LPS-induced ALI [13]. Further studies show that binding of LPS and TLR4 triggers induction of NF-κB as well as mitogen-activated protein kinase (MAPK) signal pathways, leading to severe cellular responses [4]. p38 MAPK belongs to MAPK family that is involved in a signal cascade responsive to stress stimuli [14]. And the activation of p38 MAPK has been implicated as a critical step in the process of pulmonary barrier dysfunction induced by various stimulus, including pertussis toxin, LPS, and H_2_O_2_. In addition, emerging evidences highlight role of p38 MAPK in pulmonary epithelial injury [15–19]. These data implicated the putative effect of p38 MAPK on LPS-induced epithelial injury. Hsp27 is considered to be an inhibitor of actin polymerization [20]. It has been shown to regulate actin-containing cytoskeletal structure during endothelial barrier dysfunction. Furthermore, S. Hirano et al. has presented that LPS induced endothelial barrier breakdown was associated with the phosphorylation of Hsp27 [21]. Of note, regardless of the endothelium compromised during the development of sepsis, epithelial breakdown is considered to be key mechanism underlying profound pulmonary impairment [22]. Therefore, we seek to uncover the potential role of TLR4-p38MAPK-Hsp27 signal pathway in LPS-induced pulmonary epithelial hyperpermeability.

## Methods

### Antibodies and reagents

Monoclonal antibody against F-actin was purchased from abcam. Antibodies against p38、p-p38、Hsp27 、β-actin were purchased from ABclonal. Anti-P-Hsp27 antibody was purchased from CST. TLR4 siRNA、p38 siRNA and control siRNA were synthesized by GenePharma (Shanghai, China). The p38 specific inhibitor SB203580 was obtained from MedChem Express. LPS、DAPI were acquired from Sigma.

### Cell culture

Adenocarcinomic human alveolar basal epithelial cells (A549) were cultured in 1640 medium (Gibco) supplemented with 10% fetal bovine serum (Gibco) in humidified incubator with 5% CO_2_. Experiments were performed after A549 were grown on specific plates, reaching 80%~ 90% confluences and subsequently substitute the culture medium with serum-free one for 12 h to make sure cells in synchronous growth and quiescent state.

### Isolation of mouse pulmonary epithelial cells

6–8-week-old male C57 mice, purchased from the Laboratory Animal Center of Southern Medical University, were maintained under controlled temperature (22 ± 1 °C), humidity (60 ± 10%) and light (12 h/day). All the animals were fasted for 8 h with free access to water before the experiment. All the procedures related to mice were approved by the Animal Care Committee of the Southern Medical University of China and were in strict accordance with the Guide for the Care and Use of Laboratory Animals of the National Institutes of Health.

Mice were anesthetized by an intraperitoneal injection of 1.0% pentobarbital sodium (60 mg/kg) and sacrifized through Cervical dislocation and the lungs were collected in PBS, sliced with scissors, aaand then digested with trypsin in 37 °C for 15 min. Collect the supernatant, while the tissue was further digested with 2 mL collagenase in 37 °C for 15 min. Then 70 μm nylon filter was used and the cell suspension was collected in a new tube, followed by 1200 rpm centrifugation for 5 min. Finally, cells was cultured in 37 °C 5% CO_2_ incubator.

### Cell permeability measurement

The explorations of transepithelial permeability were achieved through Transepithelial electrical resistance (TEER) measurement and testing of FITC-Dextran transepithelial flux. For TEER measurement, A549 cells were seeded on the upper chamber of 0.4 μm-pore-size-transwells (US,Coming Costar), stimulated with 100 ng/mL LPS for corresponding time, and monolayer permeability was measured with EVOM_2_ (World Precision Instruments, USA). As for FITC-Dextran transepithelial flux, cells were grown on the transwells as mentioned above, and FITC-labeled dextran (1 mg/mL) was administrated for 45 min, followed by determination of FITC-dextran concentration of upper and bottom chamber using HTS 7000 microplate reader. The permeability coefficient of dextran were calculated as follows: Pd = [A]/t × 1/A × V/[L],with [A] represents the concentration of bottom chamber, [L] represents the upper ones, t indicates time in seconds, A refers to the membrane area (in cm^2^), and V indicates the volume of the bottom chamber.

### Western blotting

Cells were cultured in 6-well plates, and treated with 100 ng/mL LPS. Total proteins were prepared in the extraction buffer containing phosphatase and protease inhibitors, and separated by 10% SDS-PAGE followed by transferred to polyvinylidenedifluoride membranes. Then, Membranes were blocked with 5% BSA and incubated with specific primary antibodies overnight at 4 °C. After three times washing, the membranes were incubated with respective second antibodies at room temperature for 1 h, and subsequently detected with chemiluminescence. Finally, analysis of band density was carried out by an imaging station.

### siRNA transfection

Cells were cultured in 6-well plates with 50% ~ 70% confluence, and transfected with pre-prepare siRNA- siRNA-Mate™ complex according to GenePharma’ s protocol. After 48 h transfection, cells were incubated with or without 100 ng/mL LPS, followed by the corresponding experiment.

### Immunofluorescent

A549 cells were seeded on Microporous Petri dish to confluence. After the indicated treatments, they were washed three times with phosphate buffered saline (PBS), fixed with 4% formaldehyde and sequentially permeabilized in 0.5% Triton X-100 at room temperature. Then cells were blocked with 5% BSA after three times wash in PBS and subjected to incubation of rhodamine-phalloidin (2000 U/L) at room temperature for 1 h, following with three times washing in PBS. Finally, DAPI (1:1000) was administrated to lable the nucleus, and Zeiss LSM780 laser confocal scanning microscope (Zeiss, Germany) was used to detect the Immunofluorescent signals.

### Animal models

One hundred 6–8-week-old male C57 mice, purchased from the Laboratory Animal Center of Southern Medical University, were used in these experiments. C57 mice were chosen because this strain is wildly used as models of sepsis. They were randomly divided into 4 groups and each group contains 3–4 mice. Afterwards, they were intraperitoneal injected with 200 μL PBS or PBS-diluted LPS (15 mg/kg) for 8 h. For p38 MAPK inhibitor group, SB203580 was administrated intraperitoneally 2 h before PBS or LPS injection. After injection, mice were kept under controlled temperature (22 ± 1 °C), humidity (60 ± 10%) and light (12 h/day). All the animals were fasted for 8 h with free access to water before the experiment.

### Measurements of acute lung injury

Mice were euthanized with 13.3% ethyl carbamate plus 0.5% chloralose intraperitoneal injection (0.65 mL/kg) after indicated treatments. The right lobes of the lung were removed and fixed with 4% paraformaldehyde for 48 h, dehydrated in graded alcohol, and embedded in paraffin, subsequently followed by hematoxylin (H) and eosin (E) staining. The total lung injury score was obtained as previously described [23]. The left lobes were removed and immediately stored in − 80 °C prior to protein analysis. Bronchoalveolar lavage Fluid (BALF) was acquired as previously described [24], with which manual cell counts were performed though hemacytometer and total protein concentration was measured using BCA protein assay. As for lung wet-to-dry weight ratio, the lungs were excised and weighted before and after dried to a constant weight at 55 °C for 72 h.

### In vivo epithelial permeability assay

Mice were going through carotid veins cannulation after they were anesthetized, and 1 mL APSS-diluted FITC-dextran (100 mg/kg) were injected through carotid veins. 30 min later, apical blood collection was performed with a heparinized syringe and the blood was centrifuged at 3000 rpm for 15 min to obtain the supernatant. BALF was acquired afterwards. BALF/blood fluorescence intensity ratio was measured with fluorospectrophotometer as indicators of epithelial permeability.

### Statistical analysis

All data were analyzed by SPSS16.0 software and presented with means ± SD with more than three independent experiments. One-way ANOVA was used in statistical comparisons with significant level set at *P* < 0.05. LSD posthoc analysis was used to compare data among multiple groups.

## Results

### In vivo study of LPS-induced pulmonary edema and epithelial hyperpermeability

**Fig. 1:**
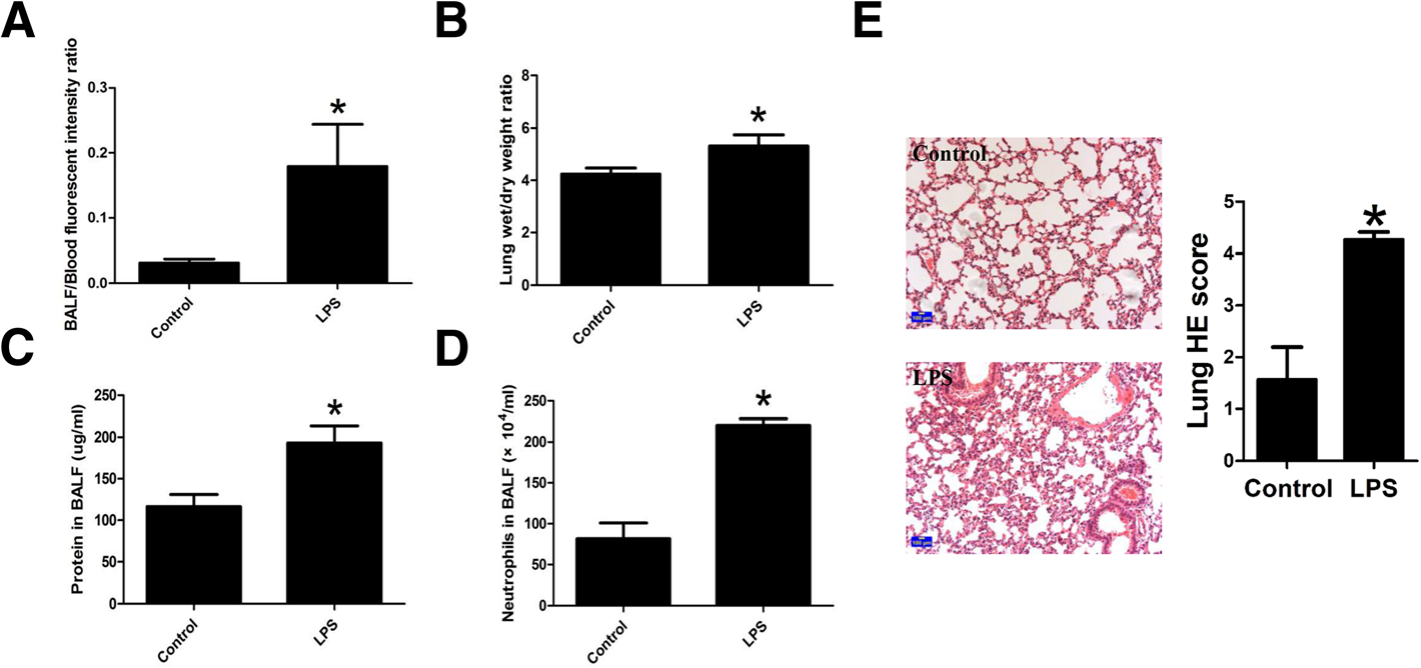
LPS induce pulmonary edema and alveolar epithelial hyperpermeability in vivo. C57 mice were intraperitoneal injected with PBS or PBS-diluted LPS (15 mg/kg) for 8 h. (**a**) BALF/blood fluorescence intensity ratio and (**b**) Wet-to-dry lung weight ratio were measured; (**c**) protein concentration and (**d**) neutrophil cell counts in BALF were obtained; *n* = 3, *P < 0.05 versus control. (**e**) Lung Hematoxylin and eosin staining were obtained and lung injury score was calculated. *n* = 5, *P < 0.05

We performed in vivo studies to determine effect of LPS in ALI, specifically in pulmonary epithelial permeability. We found that LPS-injected mice presented higher BALF/Blood fluorescent intensity ratio (Fig. 1a), implicating the breakdown of pulmonary epithelial barrier. The increased wet-to-dry lung weight ratio (Fig. 1b), and higher protein concentrations (Fig. 1c) as well as increased neutrophil cell counts in BALF (Fig. 1d) presented in LPS-treated mice implicated pulmonary edema and inflammation. Hematoxylin (H) and eosin (E) staining were performed to examine the histological changes in lung. Results showed increased lung injury score with irregular alveolar structure with thickened alveolar septa and discontinuous alveoli as well as more neutrophil infiltration in mice injected with LPS (Fig. 1e). These data indicated that alveolar epithelium dysfunction was involved in LPS-induced ALI.

### LPS induced epithelial hyperpermeability and cytoskeleton rearrangement

**Fig. 2:**
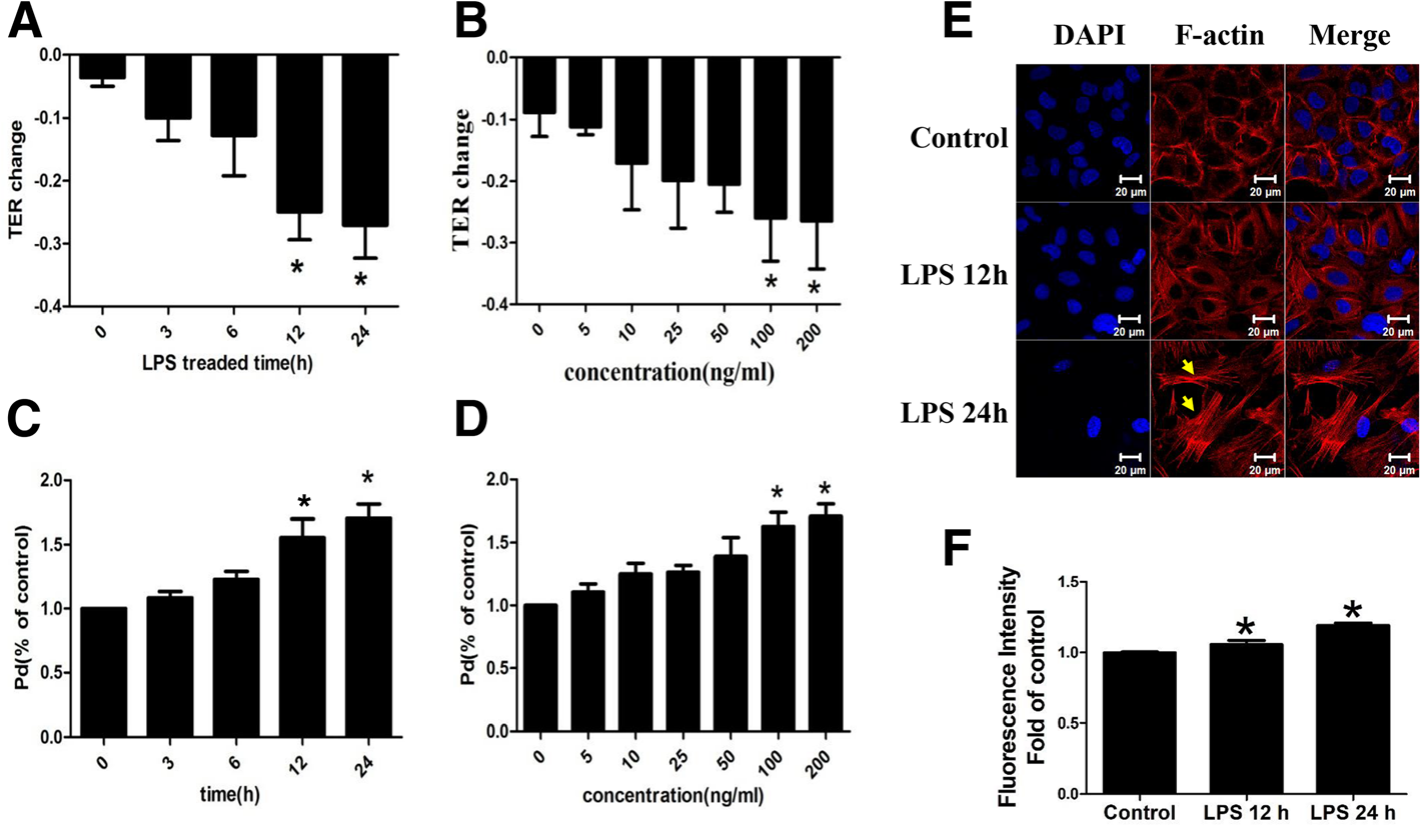
LPS induce A549 hyperpermeability and cytoskeleton rearrangement. A549 cells were stimulated by LPS in (**a**, **c**) time- or (**b**, **d**) dose-dependent manner, and (**a**, **b**) TER value and (**c**, **d**) permeability coefficient for dextran (Pd) in dextran trans-epithelial flux were evaluated. (**e**) A549 were treated with or without 100 ng/mL LPS and the image of rhodamine-phalloidin stained F-actin was obtained through a laser confocal microscopy; (**f**) Fluorescence intensity of F-actin was qualified. *n* = 3, *P < 0.05 versus control

To further confirm the role of LPS in epithelial permeability, A549 cells, which are widely used as an in vitro model for a type II pulmonary epithelial cells, were stimulated by LPS, and TEER value and permeability coefficient for dextran (Pd) in dextran trans-epithelial flux were evaluated. Data show that TEER was decreased (Fig. 2a) and Pd was gradually increased with significant differences at 12 h (Fig. 2b). When different concentrations of LPS were administrated, cells showing gradually decline trend of TEER (Fig. 2c) and incline trend of Pd (Fig. 2d) with significant differences at 100 ng/mL and 200 ng/mL. These data well confirmed that LPS could induce pulmonary epithelial hyperpermeability. Since cytoskeletal network was considered to be a key component to alveolar barrier integrity, we then monitored F-actin morphological changes to investigate whether LPS induced A549 cytoskeleton rearrangement. We found that after LPS stimulation, the periphery-located F-actin was substituted with centralized stress fibers, and the regular shaped cells with continuous intercellular connections become irregular (Fig. 2e). And the fluorescense intensity was increased after LPS administration in a time dependent manner (Fig. 2f). These data showed that cytoskeletal rearrangement was involved in LPS-induced epithelial barrier dysfunction.

### LPS induced p38 MAPK phosphorylation

**Fig. 3:**
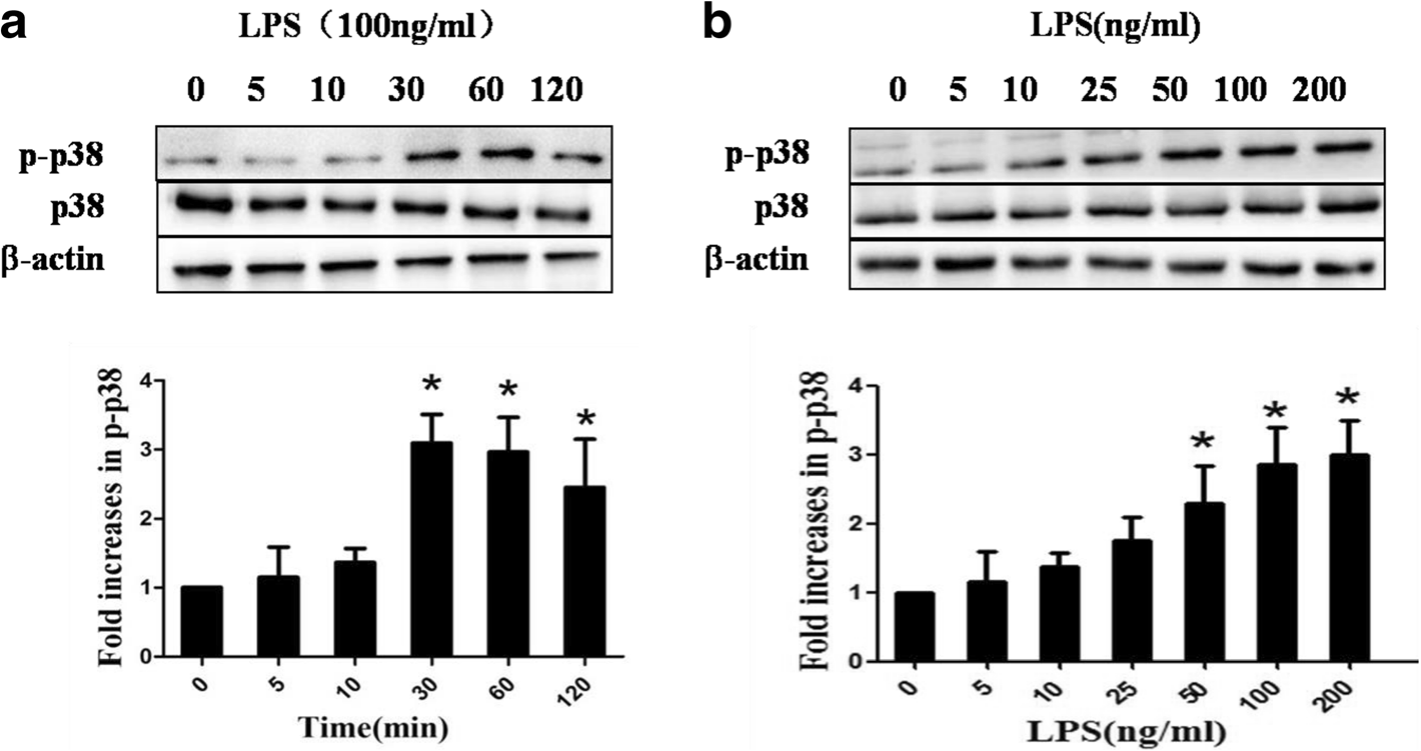
Effect of LPS on p38 MAPK phosphorylation. A549 cells were stimulated by LPS in (**a**) time- or (**b**) dose-dependent manner. p-p38 was detected by western blotting. *n* = 3, *P < 0.05 versus control

Due to the pivotal role of p38 MAPK in epithelial injury, we verified whether it is involved in LPS-induced epithelial hyperpermeability. The phosphorylation of p38 MAPK was measured after cells incubated with LPS for different time courses or at different concentrations. Results showed LPS stimulation increased p38 MAPK phosphorylation in a time-dependent manner, with reaching a peak at 30 min (Fig. 3a). Also dose-dependently enhanced p38 MAPK phosphorylation was observed. These results suggested activation of p38 MAPK was involved in LPS-mediated epithelial injury (Fig. 3b).

### Role of TLR4 in LPS-induced epithelial hyperpermeability

**Fig. 4:**
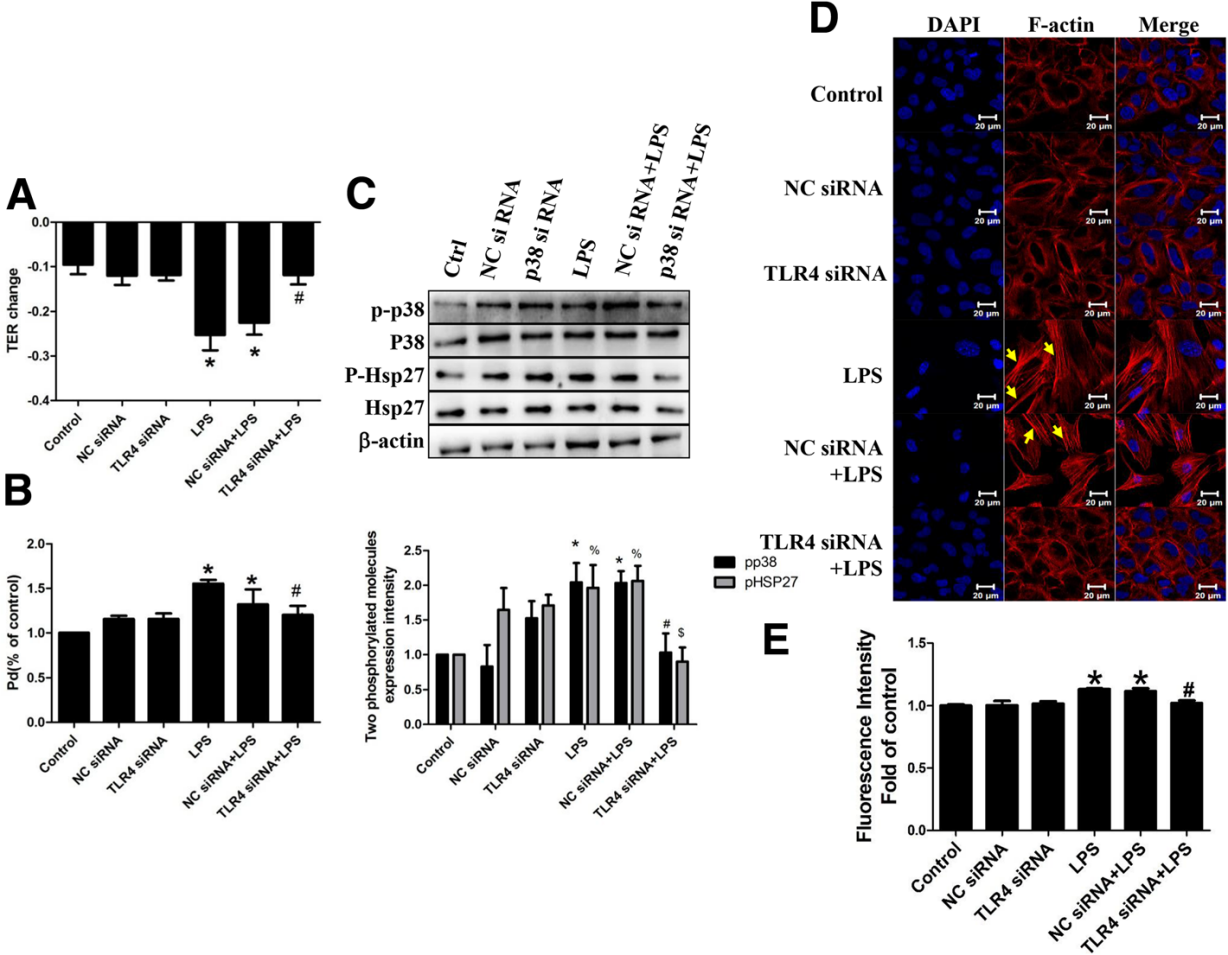
Role of TLR4 in LPS-induced A549 hyperpermeability. A549 was pretreated with TLR4 siRNA followed by LPS exposure. (**a**) TER value and (**b**) permeability coefficient for dextran (Pd) in dextran trans-epithelial flux were measured; (**c**) P-p38 and P-Hsp27 were detected by western blotting; (**d**) The image of rhodamine-phalloidin stained F-actin was obtained through a laser confocal microscopy; (**e**) Fluorescence intensity of F-actin was qualified. *n* = 3, *P < 0.05 versus control, #*P* < 0.05 versus LPS

We next explored whether TLR4 was required for p38 MAPK activation in LPS-induced epithelial hyperpermeability. We First examined that knockdown of TLR4 with siRNA significantly abolished LPS-mediated A549 hyperpermeability, rescued TEER and Pd to normal level (Fig. 4a, b). As well as the phosphorylation of p38 MAPK induced by LPS was also inhibited by TLR4 siRNA (Fig. 4c). Since Hsp27 was considered to be a downstream signal molecule of p38 MAPK, and the phosphorylation of Hsp27 was reported to be a key factor to regulate cytoskeletal alteration, we therefore investigated whether Hsp27 activation was involved in this process. Predictably, we found that LPS-mediated Hsp27 phosphorylation and F-actin rearrangement were blocked by TLR4 siRNA pretreatment (Fig. 4c, d). Fluorescence intensity of F-actin was qualified (Fig 4e).These data suggested that LPS-evoked p38MAPK-Hsp27 activation via TLR4.

### Role of p38 MAPK in LPS-induced epithelial hyperpermeability

**Fig. 5:**
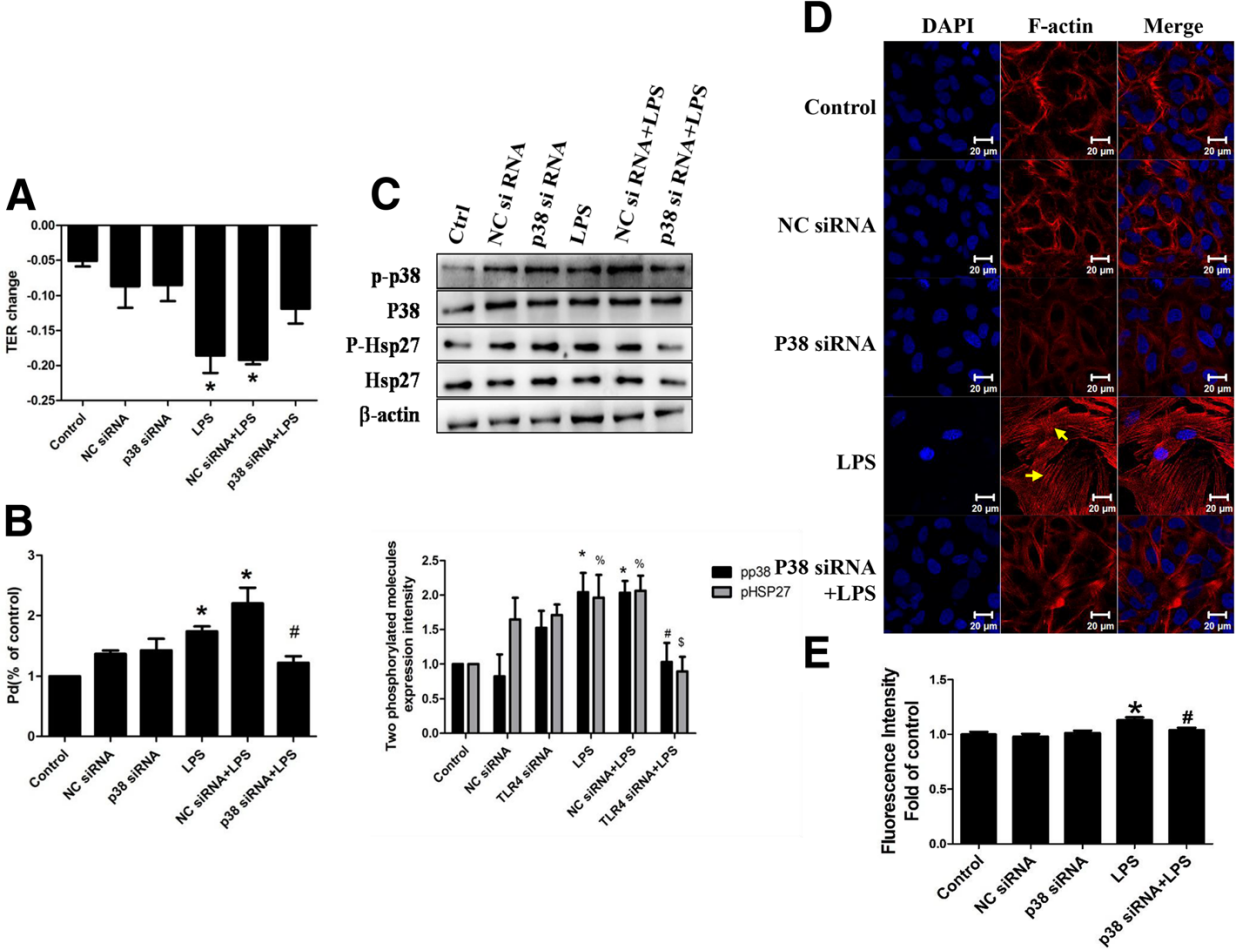
Effect of p38 siRNA in LPS-induced A549 hyperpermeability. A549 was pretransfected with p38 siRNA followed by LPS stimulation. (**a**) TER value and (**b**) permeability coefficient for dextran (Pd) in dextran trans-epithelial flux were measured; (**c**) P-p38 and P-Hsp27 were detected by western blotting; (**d**) The image of rhodamine-phalloidin stained F-actin was obtained through a laser confocal microscopy; (**e**) Fluorescence intensity of F-actin was qualified. *n* = 3, *P < 0.05 versus control, #*P* < 0.05 versus LPS

**Fig. 6:**
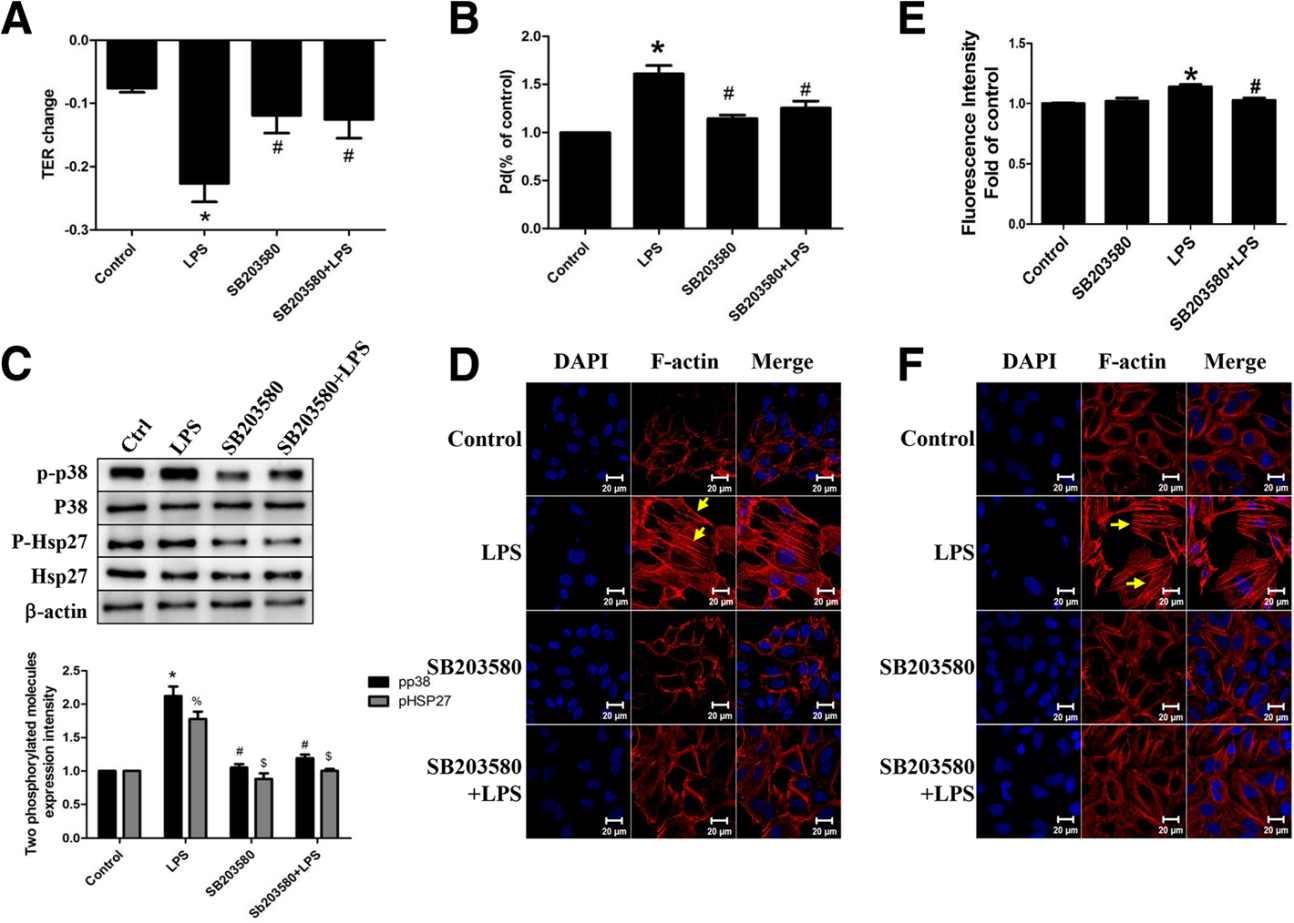
Role of p38 inhibitor SB203580 in LPS-induced A549 hyperpermeability. A549 was pretreated with SB203580 followed by LPS exposure. (**a**) TER value and (**b**) permeability coefficient for dextran (Pd) in dextran trans-epithelial flux were measured; (**c**) P-p38 and P-Hsp27 were detected by western blotting; (**d**) The image of rhodamine-phalloidin stained F-actin was obtained through a laser confocal microscopy; (**e**) Fluorescence intensity of F-actin was qualified; (**f**) The image of rhodamine-phalloidin stained F-actin was obtained through a laser confocal microscopy by using primary mice pulmonary epithelial cells.  *n* = 3, *P < 0.05 versus control, #*P* < 0.05 versus LPS

So far, we have identified that p38 MAPK was activated in response to combination of LPS and TLR4 in vitro. To further understand the role of p38 MAPK in LPS-induced epithelial barrier dysfunction, p38 MAPK siRNA or the selective inhibitor SB203580 were administrated before LPS stimulation. Results showed that p38 MAPK siRNA significantly reversed LPS-induced epithelial hyperpermeability (Fig. 5a, b). Western blotting analysis showed that p38 MAPK siRNA attenuated LPS- mediated Hsp27 phosphorylation (Fig. 5c). In addition, cells pretreated with p38 MAPK siRNA before LPS stimulation, exhibited fewer stress fibers formation compared with LPS group, indicating that p38 siRNA prevented LPS-induced F-actin remolding (Fig. 5d). Fluorescence intensity of F-actin was qualified (Fig. 5e). Accordingly, inhibition of p38 MAPK with SB203580 blocked Hsp27 phosphorylation induced by LPS (Fig. 6c), reversed LPS-mediated cytoskeleton rearrangement (Fig. 6d, e)  and thus attenuated LPS-evoked A549 hyperpermeability (Fig. 6a, b). We also examined the F-actin rearrangement induced by LPS in primary mice pulmonary epithelial cells, and got the same effects (Fig. 6f).

### In vivo study of effects of p38 MAPK in LPS-induced ALI

**Fig. 7:**
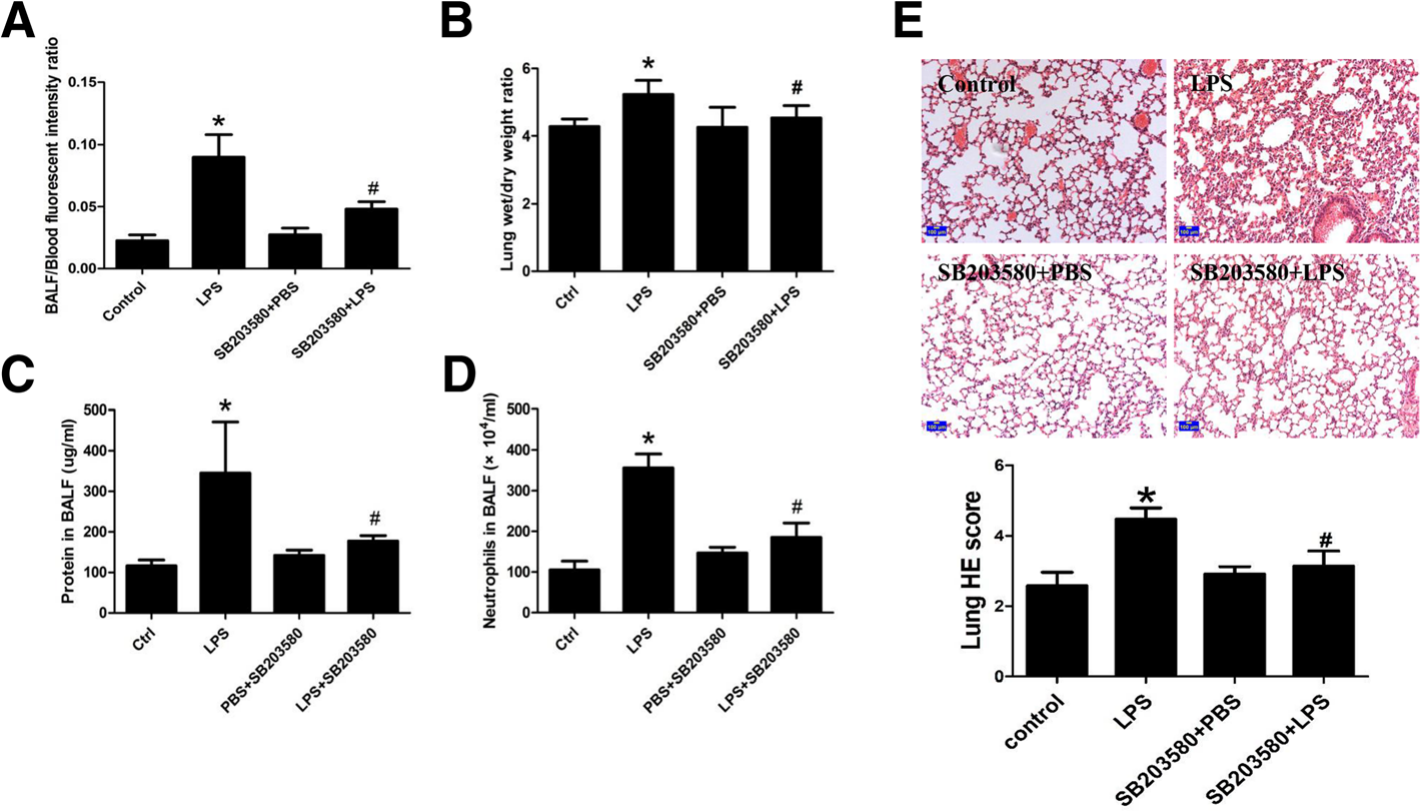
In vivo study of effect of p38 MAPK in LPS-induced ALI. C57 mice were pretreated with SB203580 before LPS (15 mg/kg) injection. (**a**) BALF/blood fluorescence intensity ratio and (**b**) Wet-to-dry lung weight ratio were measured; (**c**) protein concentration and (**d**) neutrophil cell counts in BALF were obtained; *n* = 3~ 4, *P < 0.05 versus control. (**e**) Lung Hematoxylin and eosin staining were obtained and lung injury score was calculated. *n* = 5, *P < 0.05

To further verify involvement of p38 MAPK activation in LPS-mediated ALI in vivo, p38 inhibitor SB203580 was administered before LPS injection, and the severity of ALI as well as alveolar barrier dysfunction was evaluated. Data showed that, compared with LPS group, mice pretreated with SB 203580 followed by LPS injection presented decreased wet-to-dry lung weight ratio (Fig. 7b), lower protein concentration (Fig. 7c) and less neutrophil cell counts (Fig. 7d) in BALF, and decreased BALF/blood fluorescence intensity ratio (Fig. 7a) when compared with LPS counterparts. All these observations implicated that inhibition of p38 MAPK activity significantly reversed LPS-mediated pulmonary edema and epithelial hyperpermeability. Results of Hematoxylin (H) and eosin (E) staining showed that SB203580 attenuated LPS-mediated alveolar structure disruption and neutrophil infiltration, indicated by decreased lung injury score with ameliorated irregulation of alveolar structure as well as decreased neutrophil infiltration when compared with LPS, suggesting that activation of p38 MAPK was involved in LPS-mediated ALI (Fig. 7e).

## Discussion

LPS-mediated ALI has been primarily characterized by severe edema that is caused by alveolar-capillary barrier dysfunction [25], with numerous available evidences support the critical role of pulmonary epithelium in this syndrome of respiratory failure [10, 26]. Gorin AB et al. had performed a precise study demonstrating that pulmonary epithelium accounted for more than 92% of resistance effect to albumin transit across the barrier, and that epithelial injury alone are sufficient in pulmonary edema formation [27]. This data, accompanying with other emerging evidences [11, 28] strongly spotlight pulmonary epithelium barrier dysfunction in ALI. Therefore, we highlight effect of alveolar epithelium in current study of LPS-induced ALI.

Epithelium serves as a barrier between the air-containing and the aqueous interstitial compartment. It is structurally optimized to regulate molecular transportation through trans- and paracellular ways. Early investigations shown that cytochalasin D and B, the actin altering agents, can increase epithelial permeability [29, 30], indicating the potential relationship between actin dynamics and epithelial barrier dysfunction. In addition, it has been well recognized that intracellular stress fiber formation is strongly related to cellular hyperpermeability. As reviewed by Rodgers, stress fibers resulted from actin polymerization is one of the primary elements that comprises the contractile machinery in cells and has been suggested with increased cell contractility, which is responsible for intracellular gap formation [28]. These observations beg the question of the role of cytoskeleton rearrangement in LPS-induced epithelial hyperpermeability, which remains the major theme of this study [31].

Firstly, in vivo studies were performed to investigate whether alveolar-capillary permeability was increased after mice exposure to LPS. Results presented increased wet-to-dry lung weight ratio, higher protein concentration as well as more neutrophil cell counts in BALF, higher score of total lung injury, and enhanced BALF/blood fluorescence intensity ratio, all of which indirectly or directly indicated that LPS induce breakdown of alveolar barrier integrity. This impairment was confirmed by decline of TEER and incline of Pd in dextran trans-epithelial flux in LPS-stimulated A549 cells. Afterwards, our postulation, cytoskeleton remodeling is involved in this process, was certified by morphological alteration of A549 compatible with the enhancement of stress fibers.

Sequentially, we sought to shed light on the underlying mechanism. It has been frequently implicated that p38 MAPK is either partially or totally responsible for endothelial actin polymerization during lung injury [5, 15, 32, 33]. It could also be shown that p38 MAPK mediated epithelial hyperpermeability [34]. In other cases, p38 MAPK was suggested to be cooperated with actin cytoskeleton alternation in epithelium [33]. More in-depth mechanism analysis suggest that activation of p38 MAPK was triggered by combination of LPS and TRL4 [4], and that TLR4-p38 MAPK signal pathway was involved in LPS mediated pulmonary edema formation [6]. In addition, p38 MAPK has been considered to be the upstream of Hsp27. And Hsp27 phosphrylation induced by p38 MAPK was associated with actin filament dependent endothelial hyperpermeability caused by LPS [17]. Further work showed that unphosphorylated Hsp27 binds to actin, while phosphorylated Hsp27 dissociates from actin, which facilitates actin polymerization and stress fiber formation [35]. Given those information, we naturally postulated that LPS-mediated activation of TLR4-p38 MAPK-Hsp27 signal pathway is involved in cytoskeleton rearrangement dependent alveolar epithelial hyperpermeability.

To this end, we testified that p38 was activated by LPS in A549 cells in a time- and concentration-dependent manner. Then, role of TLR4 in LPS in LPS-mediated A549 hyperpermeability was investigated by using TLR4 siRNA. Data showed that knockdown of TLR4 abolished LPS-mediated p38 and Hsp27 phosphrylation, attenuated LPS-evoked A549 hyperpermeability, and suppressed LPS-triggered cytoskeleton rearrangement, suggesting that TLR4 is involved in LPS induced pulmonary epithelial barrier dysfunction and act upstream of p38 and Hsp27. Subsequently, we testified that either genetically knockdown of p38 with siRNA or pharmacologically inhibit p38 activation with SB203580 would significantly reverse LPS-induced A549 hyperpermeability, reduce phosphorylation of Hsp27 evoked by LPS, and suppress LPS-triggered cytoskeleton rearrangement, suggesting that priming activation of p38 MAPK leads to subsequent phosphorylation of Hsp27 which in turn triggers rearrangement of actin filament and breakdown of pulmonary epithelial barrier. Afterwards, SB203580 were used in C57 mice to further confirm the pivotal role of p38 MAPK in LPS-induced ALI. Data presented decreased wet-to-dry lung weight ratio, lower protein concentration and less neutrophil cell counts in BALF, lower score of total lung injury, and decreased BALF/blood fluorescence intensity aratio, suggesting that inactivation of p38 MAPK significantly reverses LPS-induced ALI and alveolar epithelial hyperpermeability.

## Conclusion

Taken together, in the current study, we highlighted the response of alveolar epithelium to LPS-induced ALI, and presented that LPS increase alveolar epithelial permeability both in vitro and in vivo. Furthermore, TLR4- p38 MAPK- Hsp27 signal pathway was studied in an in vitro model to better understand the underlying mechanism. Additionally, we presented that inhibit p38 MAPK significantly reverse LPS-induced pulmonary epithelial dysfunction, and hence release ALI. These findings add to other promising endothelial based studies advance our comprehension of multicellular adaptation to LPS-induced ALI. The dysregulation of epithelial function during sepsis contributes to leakage of fluid and even inflammatory mediators into air-containing alveolar space, which leads to severe edema and may favor the development secondary infection. Therefore, septic alveolar epithelium should be considered either therapeutic or diagnostic targets to improve outcomes in patients.

## Abbreviations

ALI: Acute lung injury
BALF: Bronchoalveolar lavage fluid
HE: Hematoxylin and eosin
LPS: Lipopolysaccharide
PBS: Phosphate buffered saline
TEER: Transepithelial electrical resistance
